# Tuberculous meningitis is a major cause of mortality and morbidity in adults with central nervous system infections in Kota Kinabalu, Sabah, Malaysia: an observational study

**DOI:** 10.1186/s12879-016-1640-x

**Published:** 2016-06-16

**Authors:** Heng Gee Lee, Timothy William, Jayaram Menon, Anna P. Ralph, Eng Eong Ooi, Yan’an Hou, October Sessions, Tsin Wen Yeo

**Affiliations:** Department of Medicine, The Infectious Disease Unit, Queen Elizabeth Hospital, Kota Kinabalu, Sabah Malaysia; Jesselton Medical Centre, Kota Kinabalu, Sabah Malaysia; Menzies School of Health Research, Darwin, Australia; Program in Emerging Infectious Diseases, Duke-NUS Graduate Medical School, Singapore, Singapore; Lee Kong Chian School of Medicine, Nanyang Technological University, Singapore, Singapore; Communicable Disease Centre, Institute of Infectious Diseases and Epidemiology, Tan Tock Seng Hospital, Singapore, Singapore

**Keywords:** Central nervous system infections, Tuberculous meningitis, Meningitis, Encephalitis, Meningo-encephalitis

## Abstract

**Background:**

Central nervous system (CNS) infections are a significant contributor to morbidity and mortality globally. However, most published studies have been conducted in developed countries where the epidemiology and aetiology differ significantly from less developed areas. Additionally, there may be regional differences due to variation in the socio-economic levels, public health services and vaccination policies. Currently, no prospective studies have been conducted in Sabah, East Malaysia to define the epidemiology and aetiology of CNS infections. A better understanding of these is essential for the development of local guidelines for diagnosis and management.

**Methods:**

We conducted a prospective observational cohort study in patients aged 12 years and older with suspected central nervous system infections at Queen Elizabeth Hospital, Kota Kinabalu, Sabah, Malaysia between February 2012 and March 2013. Cerebrospinal fluid was sent for microscopy, biochemistry, bacterial and mycobacterial cultures, *Mycobacterium tuberculosis* polymerase chain reaction (PCR), and multiplex and MassCode PCR for various viral and bacterial pathogens.

**Results:**

A total of 84 patients with clinically suspected meningitis and encephalitis were enrolled. An aetiological agent was confirmed in 37/84 (44 %) of the patients. The most common diagnoses were tuberculous meningitis (TBM) (41/84, 48.8 %) and cryptococcal meningoencephalitis (14/84, 16.6 %). *Mycobacterium tuberculosis* was confirmed in 13/41 (31.7 %) clinically diagnosed TBM patients by cerebrospinal fluid PCR or culture. The acute case fatality rate during hospital admission was 16/84 (19 %) in all patients, 4/43 (9 %) in non-TBM, and 12/41 (29 %) in TBM patients respectively (*p* = 0.02).

**Conclusion:**

TBM is the most common cause of CNS infection in patients aged 12 years or older in Kota Kinabalu, Sabah, Malaysia and is associated with high mortality and morbidity. Further studies are required to improve the management and outcome of TBM.

## Background

Central nervous system (CNS) infections including meningitis and encephalitis result in significant morbidity and mortality in hospitalized patients [1–6]. The worldwide incidence and reported aetiology of CNS infections vary according to age, geographical region, economic status, vaccination policies and diagnostic capacity.

Prospective studies in various countries conducted to define the aetiology of CNS infections have shown contrasting results [1–6]. In an encephalitis study in England, infections (42 %) were the leading aetiology, followed by undetermined (37 %) and immune-mediated causes (21 %) [3]. Among those with infectious encephalitis, herpes simplex virus (19 %), varicella zoster virus (5 %) and *Mycobacterium tuberculosis* (MTB) (5 %) were the most common diagnoses [3]. In the California Encephalitis Project, the frequency of autoimmune N-methyl-D-aspartate receptor (anti-NMDAR) encephalitis was four times that of viral aetiologies in a cohort of young individuals [7]. In Vietnam, viral encephalitis and meningitis accounted for 34 % of adult CNS infections, with dengue (11 %), enteroviruses (10 %) and herpes simplex (11 %) being the most commonly identified pathogens, while tuberculous meningitis (TBM) accounted for 14 % of cases [5]. S*treptococcus pneumoniae* and *Neisseria meningitides* are the most common pathogens of bacterial meningitis in developed countries, however *Streptococcus suis*, a zoonotic pathogens from pigs, is the most common cause in Vietnam [4, 5]. Taken together, these data suggest that local data are required to develop clinically relevant guidelines

In Malaysia, only confirmed cases of viral encephalitis are notifiable while meningitis is not, requiring prospective studies to detail the epidemiology of CNS infections. In Sarawak, East Malaysia, paediatric studies of CNS Japanese encephalitis and human enterovirus 71 infections have been undertaken [8, 9]. However, no prospective studies have been conducted in Malaysian Borneo, to define the epidemiology and aetiology of meningitis and encephalitis in adults. In anticipation of future development of local clinical guidelines, we conducted a prospective hospital-based study to evaluate the aetiology and epidemiology of CNS infections in Kota Kinabalu, Sabah, Malaysia.

## Methods

### Study site and design

This was a prospective observational cohort study conducted at Queen Elizabeth Hospital, Kota Kinabalu, Sabah, East Malaysia, an adult tertiary care hospital with a catchment population of over 1 million. We screened all inpatients with suspected meningitis or encephalitis from February 2012 till March 2013. Research clinicians used a standardised data sheet to collect demographic data, epidemiological information, clinical history, vital signs, physical observations, biochemical and microbiological data, imaging reports, treatment, outcome, and final diagnosis. The research team followed up patients regularly until discharge or death. All patients were managed according to Malaysian Ministry of Health guidelines. For TBM, in addition to anti-tubercular medication, this included dexamethasone 0.3–0.4 mg/kg/day for week 1, 0.2–0.3 mg/kg/day for week 2, 0.1–0.2 mg/kg/day for week 3 and 0.1 mg/kg/day for week 4, followed by 4 weeks of dexamethasone decreasing by 1 mg each week. Childhood vaccinations for potential CNS pathogens in Sabah, Malaysia include diphtheria-pertussis-tetanus, measles-mumps-rubella, polio, Bacillus Calmette-Guerin (BCG) and *Haemophilus influenza type B* vaccine. In Malaysia, HIV screening is performed only for pathogens and infections associated with HIV including *Mycobaterium tuberculosis, Cryptococcus* species and syphilis but is not routine.

### Definitions of clinical syndromes and outcome

Meningitis was defined as cerebrospinal fluid (CSF) pleocytosis (≥5 WBC/μL) plus two or more of the following: i) fever or history of fever (≥38^0^C), ii) neck stiffness, iii) headache, and was further classified as acute if symptoms were present ≤ 7 days or chronic if longer.

Encephalitis was defined as encephalopathy (altered level of consciousness, lethargy, irritability, and change in behaviour or personality) longer than 24 h with two or more of the following: i) fever or history of fever (≥38^0^C), ii) CSF pleocytosis (≥5 WBC/μL), iii) seizures or focal neurological findings, iv) abnormal neuroimaging consistent with encephalitis or v) abnormal electroencephalogram findings compatible with encephalitis. Patients with neck stiffness and encephalitis were defined as meningoencephalitis. A published consensus case definition was used to classify TBM into confirmed, probable and possible cases [10].

Criteria for exclusion were non-infectious CNS disorders due to hypoxic, vascular, toxic and metabolic causes, patients with CNS disorders lasting less than 24 h, and patients with malaria diagnosed on microscopy. Patients with an immune mediated post-infectious aetiology such as acute disseminated encephalomyelitis (ADEM) were enrolled as these often were associated with a parainfectious aetiology. We used the modified Rankin score (0 Asymptomatic, 1 Symptomatic; but no significant disability, and able to carry out all usual activities and duties, 2 Mild disability; able to function without assistance but unable to perform all previous activities, 3 Moderate disability; walk without assistance but needs help for some activities, 4 Moderately severe disability; unable to walk without assistance and attend to bodily functions, 5 Severe disability; bedridden and requiring constant care, 6 Death) to categorize the neurological status of the study subjects at the time of discharge from the hospital.

### Laboratory and microbiological study procedure

Blood and CSF samples collected as part of clinical care were used for routine and research investigations. Blood was sent for bacterial cultures, malaria screening by microscopy, rapid plasma reagin (RPR) and HIV testing if indicated as mentioned above. In addition to this, a convalescent blood, nasopharyngeal and throat swab, and rectal swab were obtained if indicated. Routine CSF testing involved (i) Gram stain for bacteria, (ii) Ziehl-Nielsen stain for mycobacteria, (iii) India ink stain and cryptococcal antigen for cryptococcus, (iv) bacterial culture, (v) glucose and total protein, and (vi) total and differential white cell count. For suspected TBM cases, 1-6 ml of CSF was sent for MTB PCR and/or culture at the Sabah State Public Health Laboratory. Anti-NMDAR antibodies were measured with a semi-quantitative indirect fluorescent antibody method at a local laboratory. CSF analysis for Japanese encephalitis IgM was performed at the Malaysian National Public Health Laboratory at Sungai Buloh, Peninsular Malaysia.

### Molecular testing procedures

A standardised and stratified approach was used with first line testing on CSF samples using RT-PCR in a research laboratory for viral pathogens as detailed in Table 1. Negative results were followed by second line testing using the MassCode (Agilent) platform.

**Table 1:** Molecular Tests Conducted on CSF Samples

	Viral pathogens	Bacterial pathogens
First Level Tests Done for CSF samples	(i) herpes simplex virus 1 and 2, (ii) varicella zoster virus, (iii) enterovirus, (iv) Japanese encephalitis virus, (v) dengue virus, (vi) nipah virus, (vii) cytomegalovirus, (viii) West Nile virus, and (ix) bunyavirus	Not Done
Second Level Test Done for CSF samples	cytomegalovirus, Epstein Barr virus, La Crosse Virus, mumps, varicella zoster virus, measles,, human herpes virus 6, rabies, LCMV, California encephalitis group (Bunyavirus), Human Alphavirus group (Eastern, Western, Venezualan EEV), Bartonella, Flavivirus group, Enterovirus group,, St. Louis Encephalitis virus and West Nile Virus	*Haemophilus influenza, Listeria monocytogenes, Mycoplasma pneumoniae, Streptococcus pneumoniae, Staphylococcus aureus, Streptococcus agalactiae, Neisseria meningitis, Rickettsia, Leptospira, Borrelia, Legionella pneumoniae, Mycobacterium tuberculosis*

In brief, nucleic acid from CSF was extracted using the QIAmp Viral RNA mini kit. Complementary DNA synthesis, PCR and purification of amplicons were done using Agilent’s MassCode reagents. MassCode is a form of multiplex PCR, which utilizes a ‘tag’ with a distinct molecular mass conjugated directly to PCR primers [11]. Upon amplification of a pathogen sequence, these masses are then incorporated into the resulting amplicons. Masses are then cleaved off the amplicons and identified via mass spectrometry. This methodology allows for rapid and simultaneous screening against large panels of known pathogens using minimal amount of sample [12, 13]. The list of pathogens tested for using this method are detailed in Table 1.

### Statistical analysis

Patients were classified according to clinical syndrome and the final aetiological diagnosis. Continuous variables were summarized as medians and range and differences between groups were analysed using Kruskal-Wallis test. Categorical variables were summarized as numbers and percentages and differences between groups compared using chi-squared or Fisher’s exact test. All analyses were performed using Stata version 12 (StataCorp, College Station, Texas, USA). A two-sided value of *p* < 0.05 was considered significant.

### Ethics statement

The study was approved by the Medical Research Sub-Committee of the Malaysian Ministry of Health (Reference NMRR-11-623-9629) and Menzies School of Health Research, Australia (Reference 2011-1636). All data were anonymised prior to analysis. Informed consent was obtained from patients or available relatives if their neurological condition did not allow them to. Informed consent was additionally obtained from these patients upon recovery.

## Results

### Baseline characteristics

There were 92 patients screened for the study and eight were excluded with alternative diagnoses (1 septic encephalopathy, one hypoxic encephalopathy, one cerebellar tumour, two acute psychosis, one cerebrovascular accident, one pneumonia, one suspected prion disease). The median age was 32.5 (range 13–67) years, 60 (71 %) were male, 15(18 %) HIV positive, and 55 (65 %) had no significant past medical history. Of the HIV patients, 11 had CD4 counts of <50/ml, 1 had 50/ml, and three had between 100–200/ml. Patients were from 18 of 23 districts in Sabah; 34/84 (40.5 %) were from the Kota Kinabalu district and 63/84 (75 %) from the urban West Coast Division of Sabah. 68/84 (81 %) patients were Malaysian; with 22/84 (26.2 %) and 13/84 (15.5 %) from the majority Kadazan-Dusun and Bajau ethnic groups respectively.

### Clinical syndromes and aetiological diagnosis

Patients were categorized into the following clinical syndromes; acute meningitis (*n* = 10: viral = 2, bacterial = 1, fungal = 4, unidentified = 3), chronic meningitis (*n* = 9: all *Cryptococcus sp*), meningoencephalitis (*n* = 12: viral = 1, fungal = 1, parasitic = 1, neurosyphilis = 2, unidentified = 7), encephalitis (*n* = 12: viral = 2, parasitic = 3, autoimmune encephalitis = 1, ADEM = 1, unidentified = 5), confirmed tuberculous meningitis (*n* = 13), probable tuberculous meningitis (*n* = 20), and possible tuberculous meningitis (*n* = 8) (Table 2). The demographic details, vital signs, laboratory results and cerebrospinal fluid results of these patients are summarized in Table 2.

**Table 2:** Demographic, vitals signs, laboratory and cerebrospinal fluid results on enrolment

	Tuberculous meningitis	Acute meningitis	Chronic meningitis	Encephalitis	Meningoencephalitis	*P* values
Number	41	10	9	12	12	
Demographic results						
Age; years, (median, range)	31 (13–64)	33 (15–65)	36.5 (22–67)	38 (16–57)	36 (14–62)	*p* = 0.8
Males; (number, percentage)	31 (75.6 %)	6 (66.6 %)	7 (77.7 %)	9 (75 %)	6 (50 %)	*p* = 0.4
Fever History (number, percentage)	32 (78 %)	9 (100 %)	7 (77.8 %)	10 (83.3 %)	8 (66.6 %)	*p* = 0.4
Days of fever before presentation (median, range)	14 (3–90)	3 (1–19)	35 (7–90)	5 (2–21)	4 (2–10)	*p* < 0.001
Headache	33 (80.5 %)	8 (88.9 %)	8 (88.9 %)	8 (66.6 %)	7 (63.6 %)	*p* = 0.6
Coma (GCS < 11)	12 (29.2 %)	2 (22.2 %)	0 (0 %)	4 (33.3 %)	3 (25 %)	*p* = 0.4
HIV Positive (number, percentage)	4 (9.8 %)	3 (33.3 %)	4 (44.4 %)	2 (16.6 %)	3 (27.7 %)	*p* = 0.3
Vitals signs and laboratory results						
Pulse Rate; median (range), (beats/min)	85 (55–155)	101 (50–160)	88 (68–108)	101 (65–140)	99 (60–162)	*p* = 0.2
Temperature; mean (range) (degrees Celsius)	37.8 (36–40)	38.5 (36.5–39)	37.6 (36.2–38.2)	38 (36.6–40.8)	36.8 (36.8–39.3)	*p* = 0.4
White blood cell count; mean (range), ×10^3^/μL	10.3 (4.3–25.3)	10.8 (6–30.3)	9.8 (4.3–13)	15.2 (4.1 to 24.9)	10 (4–22.2)	*p* = 0.3
Hemoglobin; mean (range), (g/dl)	12.4 (8.1–16.8)	13.4 (7.4–15.9)	12.1 (8–16.4)	12 (8.2–15.9)	12.1 (8.8–15.3)	*p* = 0.8
Platelet; mean (range), ×10^9^/L	314 (83–582)	251 (86–296)	274 (212–443)	193 (58–767)	299 (61–470)	*p* = 0.1
Lumbar Puncture Results						
Opening Pressure (cm H_2_O)	21 (8–65)	6 (4–27)	17 (4–40)	15 (8–40)	15 (3–34)	*p* = 0.2
Total White Cell Count	60 (0–1160)	95 (20–220)	40 (5–1050)	50 (8–615)	30 (0–305)	*p* = 0.4
Neutrophil (Percentage)	5 (0–92)	20 (5–89)	30 (0–99)	10 (0–100)	4 (0–30)	*p* = 0.09
>50 % Neutrophils (number, percentage)	7 (17.1 %)	2 (22.2 %)	3 (33.3 %)	5 (41.7 %)	6 (50 %)	*p* = 0.04
Glucose (mmol/L)	2 (0.2-4.6)	3 (0.1-6)	1.7 (0.2-3)	3.8 (0.5-6.2)	4 (2-5)	*p* = 0.7
CSF:blood glucose ratio	0.26 (0.09–0.51)	0.45 (0.05–0.87)	0.17 (0.1–0.31)	0.55 (0.06–0.83)	0.64 (0.32–0.8)	*p* < 0.001
CSF:blood glucose ratio <50 % (number, percentage)	37 (90.2)	5 (55.5)	8 (88.8)	4 (33.3)	2 (18.2)	*p* = 0.03
Total Protein g/L	1.6 (0.3–7.1)	1.3 (0.5–4.2)	1.2 (0.7–3.8)	0.9 (0.4–8.3)	0.95 (0.3–1.6)	*p* = 0.06

*P* < .05 by analysis of variance, the Kruskal-Wallis tests, *χ* ^2^ test or Fisher’s exact test comparing the groups

A confirmed aetiological diagnosis was made in 37 of 84 (44 %) patients, with *Mycobacterium tuberculosis* (13/84, 15.5 %) the most common confirmed pathogen (Table 3). In the 43 patients who did not have confirmed, probable or possible TBM, the following aetiologies were: viral (*n* = 5, (3 enterovirus, 1 Epstein Barr virus, 1 Japanese encephalitis), bacterial (*n* = 1, *Streptococcus pneumonia*), fungal (*n* = 14, all *Cryptococcus species*), parasitic (*n* = 4, (3 *Toxoplasma* sp, 1 *Naegleria fowleri*), neurosyphilis (*n* = 2), anti-NMDAR encephalitis (*n* = 1) and acute disseminated encephalomyelitis (ADEM) (*n* = 1), while 15 remained unidentified (Table 3). No cases of herpes simplex virus meningitis or encephalitis were diagnosed.

**Table 3:** Classification and causes of CNS infections

Aetiology	Total (%)
Infectious Cause	n=67 (80%)
*Mycobacterium tuberculosis* (Confirmed, probable and possible)	41 (50 %)
*Cryptococcus sp*	14 (15 %)
*Treponema pallidum*	2 (2 %)
*Toxoplasma gondii*	3 (4 %)
Japanese Encephalitis virus	1 (1 %)
Epstein–Barr virus	1 (1 %)
*Naegleria fowleri*	1 (1 %)
*Enterovirus*	3
*Streptococcus pneumoniae*	1
Immune-mediated cause	n = 2 (2 %)
ADEM	1 (1 %)
NMDAR Encephalitis	1 (1 %)
Unidentified	n = 15 (23 %)
Total	n = 84

TBM was the clinical diagnosis in 41/84 (48.8 %) of cases with CSF sent for MTB culture and PCR in 59 patients. Of those with clinical TBM, 13 of 41 (32 %) were confirmed by MTB PCR and four of this 13 were additionally MTB culture-positive (overall culture-positive rate 4/41 (9.8 %); none were culture-positive but PCR negative and no non-TBM patient was PCR or culture positive for MTB. The remaining 18/41 (43.9 %) patients had probable TBM and 11/41 (26.8 %) possible TBM using the consensus case definition [10]. Four of 41 (9.8 %) patients with TBM had HIV and 37 (90.5 %) were HIV negative and apparently immunocompetent (Table 4).

**Table 4:** Causes of CNS infections in immunocompetent versus immunocompromised patients

Aetiology	Immunocompetent^a^ *n* = 64 (76 %)	Immunocompromised^b^ *n* = 20 (24 %)	Total *n* = 84
*Mycobacterium tuberculosis*	37	4	41
*Cryptococcus sp*	5	9	14
*Toxoplasma gondii*	-	3	3
*Treponema pallidum*	1	1	2
Japanese Encephalitis virus	1	-	1
Epstein–Barr virus	1	-	1
*Naegleria fowleri*	1	-	1
ADEM	1	-	1
NMDAR Encephalitis	1	-	1
Unidentified	12	3	15

^a^Includes cases for whom immune status was unknown

^b^Reasons for immunocompromised status: 15 HIV positive; 3 on steroid therapy; 1 post bone marrow transplant; 1 post splenectomy

Cryptococcal meningoencephalitis was the second commonest CNS infection (14/84, 16.6 %) diagnosed by cryptococcal antigen detection, with fungal culture and speciation unavailable. In this group, 9/14 (64 %) patients had HIV or other immunodeficiencies while 5 had no apparent immunodeficiency (Table 4). There were two cases (2.4 %) of confirmed neurosyphilis, three cases (3.6 %) of probable cerebral toxoplasmosis in HIV patients, three patients with enterovirus infection and one case (1.2 %) each of confirmed Japanese encephalitis (CSF Japanese encephalitis IgM positive) and Epstein-Barr virus meningitis. There was a case of probable primary amoebic meningoencephalitis (PAM) due to *Naegleria fowleri* diagnosed by CSF wet mount examination. Besides infectious aetiologies, we diagnosed one case each of acute disseminated encephalomyelitis (ADEM) and anti-N-methyl D-aspartate receptor (anti-NMDAR) encephalitis. There were 15 patients with no aetiological diagnosis after initial microbiological testing and who did not have a clinical diagnosis of TBM. In these patients, cerebrospinal fluid was available for extended molecular testing, but no additional aetiological agent was identified. The use of the MassCode platform did not result in the detection of any new pathogen not previously diagnosed.

### Outcome

The overall acute case fatality rate was 19 % (16/84) during hospital admission. Additionally, 4 % (3/84), 15 % (13/84) and 20 % (17/84) had severe, moderately severe, or moderate neurological disability respectively on discharge. Five percent (4/84) had slight or no significant disability and 37 % (31/84) made a full recovery. The duration of hospital stay was prolonged in all categories, with a median of 28 days (range 4–59) in acute meningitis, 33 days (range 22–80) for chronic meningitis, 22 days (range 12-72) for encephalitis and 24 days (range 1-280) for confirmed, probable and possible tuberculous meningitis.

The outcome from TBM was significantly worse than non-tuberculous causes, with respective mortality rates of 29 % (12/41) versus 9 % (4/43) (*p* = 0.02), and severe or moderately-severe neurological deficits of 27 % (11/41) versus 11 % (5/43) (*p* = 0.04). All TBM patients received corticosteroids and anti-tubercular medications. In TBM patients, there was no significant difference in the median time to start of specific anti-tubercular medication in the patients who died or were severely disabled (2.5 days (range 0–4)) compared to those who had a good outcome (2 days (range 1–3)). In the TBM patients who died, nine were within 30 days; eight from presumed hydrocephalus and extensive cerebral infarctions, and one from multiple tuberculomas with brainstem herniation, while 3 others expired at 52, 63 and 140 days after admission. In patients with cryptococcal meningitis, 14 % (2/14) patients had a fatal outcome while 21 % (3/14) were severely disabled. In the 15 patients without TBM and no aetiological agent identified, there were two deaths (13.3 %), while 12 (80 %) made a full or good recovery. The other two patients with a fatal outcome included one with Japanese encephalitis and another with HIV and toxoplasmosis.

## Discussion

In this prospective study of adult CNS infection in a tertiary hospital in Kota Kinabalu, Sabah, East Malaysia, an aetiological diagnosis was confirmed in 44 % of patients. Whether classified according to clinical syndrome or a confirmed aetiology, tuberculous meningitis (TBM) was the most common diagnosis, accounting for almost 50 % of CNS infections. Compared to other diagnoses, TBM was associated with a significantly higher proportion of death and severe neurological disability. TB is well-recognized as a major problem in East Malaysia, but rates of drug resistance and HIV in the community are fortunately low [14, 15]. For all categories of central nervous system infections, the duration of hospital stay was also fairly long, probably reflecting the time for neurological sequelae to resolve or stabilize. The aetiological agents identified in this study contrast with those previously described in developed countries including England, USA and France, where viral infections and autoimmune encephalitis were the major diagnoses [1–3]. These differences may not be surprising due to geographic, climatic and economic differences. However, our results also differ compared to studies conducted from the South and East Asian countries of Nepal and Vietnam [4–6], highlighting the importance of local data.

The diagnosis of TBM remains challenging despite availability of new diagnostic tools [16]. In this cohort, only 32 % of patients clinically classified as TBM according to a consensus criteria had a microbiological diagnosis. All were confirmed by MTB PCR with a smaller proportion also culture-positive, while no patient had CSF acid-fast bacilli observed by microscopy using the Ziehl-Neelsen stain. These and other published results suggest novel diagnostic methods with increased sensitivity are required to further improve the diagnosis of TBM [16]. Over half of TB meningitis patients had either a fatal outcome or severe neurological deficits despite institution of anti-tubercular therapy and steroids; a finding similar to other studies [17]. A recent large well-powered study of intensified dose rifampicin for TBM did not result in an improvement in outcome [18], and adjunctive agents will be required to improve the poor outcome.

Cryptococcal sp. was the major cause of CNS infection in immunocompromised patients (Table 4), emphasising the importance of testing for Cryptococcus in this group using serum/CSF cryptococcal antigen and/or Indian ink. However, 5/14 patients diagnosed with cryptococcal meningoencephalitis were immunocompetent. *Cryptococcus gatti* and *Cryptococcus neoformans var grubii* CNS infections are well-recognized in apparently immunocompetent hosts and have been reported in Vietnam and Malaysia [19–21]. However local microbiological facilities were not able to differentiate the cryptococcal species and we did not comprehensively exclude obscure forms of immunosuppression. We speculate that some of these cases were likely to be *Cryptococcus gatti.* This has clinical importance since longer treatment durations of up to 18 months and worse neurological outcomes are reported in *Cryptococcus gatti* infections [19, 20]. Similar to previous studies, cryptococcal meningitis was associated with a poor outcome with approximately one third of diagnosed patients dead or disabled.

In non-TB, non-cryptococcal meningitis, the proportion of microbiologically confirmed diagnoses was low. Only one patient had a bacterial pathogen isolated from CSF by culture, which may be due to the high rate of outpatient antibiotic use. Bacterial PCR may be more sensitive in detecting pathogens after antibiotic use, but no additional bacterial pathogens were detected using this, and more studies are needed to further evaluate the utility in this setting. The availability of viral PCR allowed us to confirm a viral diagnosis in several patients. Finally, clinicians need to be aware of rare but serious causes of CNS infection and inflammation in Sabah, Malaysia. Identification of single patients with ADEM, anti-NMDAR encephalitis and primary amoebic meningoencephalitis highlight the broad spectrum of aetiologies requiring consideration. This is of more than academic importance as the treatment strategies are radically different. The patients with ADEM and anti-NMDAR encephalitis were managed by immunomodulation using steroids in the former, and intravenous immunoglobulins and steroids in the latter. The patient with amoebic meningoencephalitis survived after receiving a combination of amphotericin B, fluconazole and rifampicin. Despite the use of modern molecular methods, almost 60 per cent of the aetiological agents responsible for central nervous infections in our study remained unidentified. This is comparable to previously-mentioned studies conducted in England, France, USA and Vietnam. However, non-identification of a confirmed pathogen was not associated with increased mortality compared to TBM or the fatality rates observed in the aforementioned studies.

## Conclusion

Tuberculous meningitis was the most common diagnosis in a prospective study of CNS infections in an adult tertiary hospital in Sabah, East Malaysia, and was associated with high rates of death and severe neurological disability. Central nervous system involvement is the most serious complication of *Mycobacterium tuberculosis* infection, and further research is required to improve the diagnosis and outcome of this devastating disease.

## Abbreviations

ADEM, acute disseminated encephalomyelitis; CNS, central nervous system; CSF, cerebrospinal fluid; HIV, human immunodeficiency virus; MTB, *mycobacterium tuberculosis;* NMDAR, N-methyl D-aspartate; PCR, polymerase chain reaction; TBM, tuberculous meningitis
